# Repeat Keratoplasty for Failed Therapeutic Keratoplasty for Microbial Keratitis: An Analysis of Characteristics and Risk Factors

**DOI:** 10.1155/2020/9041837

**Published:** 2020-02-11

**Authors:** Jifeng Wan, Jing Lin, Yin Hu, Menghuan Wei, Yingshi Zou, Zhaohui Yuan

**Affiliations:** State Key Laboratory of Ophthalmology, Zhongshan Opthalmic Center, Sun Yat-Sen University, Guangzhou, China

## Abstract

**Purpose:**

To report the ratio of repeat-to-initial keratoplasty among patients who had underwent therapeutic keratoplasty for microbial keratitis in Southern China and to investigate the characteristics and risk factors of repeat keratoplasty.

**Methods:**

A retrospective and inclusive review of the clinical records of patients who had received therapeutic keratoplasty for microbial keratitis, at Zhongshan Ophthalmic Center during December 2012 to January 2018, was performed. Patients who suffered coexistent endophthalmitis or underwent keratoplasty combined with other surgeries were excluded. Data on clinical characteristics of all eligible patients were collected.

**Results:**

A total of 447 patients were identified. Their mean age was 48.7 ± 15.5 years, and 290 (64.9%) were male. Out of the 447 patients, 18 (4.0%) received repeat keratoplasty. Their mean age was 45.9 ± 11.3 years, and 14 (77.8%) were male. The most common indication of repeat keratoplasty (12/18) was refractory infectious keratitis. Most of the patients (15/18, 83.3%) received the second keratoplasty within 12 months after the initial keratoplasty. Factors, including age, gender, initial causative organism, presence of initial corneal perforation, ocular comorbidities, and surgical procedures were not found statistically significantly different between patients who received and not received repeat keratoplasty.

**Conclusion:**

The ratio of repeat-to-initial keratoplasty for therapeutic keratoplasty is low, compared to a failure rate of the initial grafts of over 50% reported in previous studies. The low ratio and the most common indication of repeat keratoplasty, refractory infectious keratitis, reflect caution for performing regrafts in such patients.

## 1. Instruction

Microbial keratitis is a serious disease and a common cause of blindness. Although in recent decades the availability of potent antimicrobial drugs has improved the chance for medical control of the disease [1–6], there are still progressive cases refractory to maximal medical therapy [7–9]. Therapeutic keratoplasty is a valuable surgical strategy for medically uncontrolled microbial keratitis. The primary goal of the surgery is to eradicate the infection and restore the ocular structure. Anatomic outcomes of therapeutic keratoplasty are satisfactory, although visual outcomes in most reports have been dismal [10, 11].

Repeat keratoplasty is of great significance for patients who have underwent therapeutic keratoplasty but with recurrence of the primary infection or a new infection that are medically nonresponsive. Moreover, repeat keratoplasty offers viable options for visual rehabilitation of patients with failed therapeutic keratoplasty. It has been reported that regraft after therapeutic keratoplasty could achieve long-term graft survival and the visual outcomes were relatively good [12, 13].

To achieve optimal visual rehabilitation, repeat keratoplasty may be needed in up to 50% of the cases who have underwent therapeutic keratoplasty [10, 11, 14]. However, a study conducted in Arabia has reported that the repeat-to-initial keratoplasty ratio was only 3.0% [14]. Because of the paucity of relevant studies, little is known about the rate of repeat keratoplasty for failed therapeutic keratoplasty in the real-world clinical practice, nor are the characteristics and risk factors well documented.

This study reviewed the characteristics, including the ratio of repeat-to-initial keratoplasty, indications, time intervals between the two keratoplasties, surgical procedures, and characteristics of initial microbial keratitis, of repeat keratoplasty performed for failed therapeutic keratoplasty at a tertiary eye care center in southern China. Potential risk factors of repeat keratoplasty were also explored.

## 2. Materials and Methods

### 2.1. Study Design

This was a retrospective and inclusive case series of patients who received therapeutic keratoplasty for medically refractory microbial keratitis from December 2012 to January 2018 at Zhongshan Ophthalmic Center, Guangzhou, China. All eligible patients diagnosed with bacterial, fungal, acanthamoeba, or herpes simplex keratitis were included. Patients who suffered coexistent endophthalmitis or underwent keratoplasty combined with other surgeries were excluded. The study was approved by the Ethics Committee of the Zhongshan Ophthalmic Center, Sun Yat-sen University, Guangzhou, and conducted in accordance with the Declaration of Helsinki.

Clinical records of all eligible patients were reviewed. Data obtained from medical records included: (1) diagnosis, (2) causative organism(s), (3) characteristics of the keratitis (including the size and depth of the lesion, as well as the presence of corneal perforation), (4) indication of surgery, (5) type of surgery, (6) time interval from the initial to repeat keratoplasty if applicable, (7) presence or medical history of other ocular conditions before the surgery, and (8) visual acuity and treatments after the surgery.

### 2.2. Microbiological Tests and Surgical Techniques

On presentation and at time of the initial therapeutic keratoplasty, corneal scrapings of infected lesions were performed to obtain smears and cultures and to identify microbial organisms. Additionally, antimicrobial susceptibility testing was performed on bacterial and fungal isolates. For patients who needed a repeat therapeutic keratoplasty for recurrence of the primary infection or a new infection, microbiological tests and antimicrobial susceptibility test were also administrated.

A total of seven surgeons performed keratoplasty. All the corneal buttons in this study were sourced from Zhongshan Ophthalmic Center Eye Bank. Lamellar keratoplasty or penetrating keratoplasty were performed for all cases. Surgical techniques varied according to the size, severity, and stromal depth of corneal lesions. Lamellar keratoplasty was chosen if lesion appeared not to breach the Descemet membrane. Otherwise, penetrating keratoplasty would be performed. Routine keratoplasty was performed with a graft diameter of 0.25 mm or 0.5 mm larger than the graft bed. The grafts were sutured with interrupted 10–0 nylon sutures. No curettage was performed on corneal epithelium, and corneal epithelial drying was prevented throughout the surgery.

### 2.3. Postoperative Treatment

Postoperatively, the patients were treated with topical ganciclovir, natamycin, antibiotics, or metronidazole based on the causative organisms of their initial keratitis. Patients who suffered bacterial or viral keratitis were treated with 1% prednisolone acetate for 3 weeks, later changed to 0.1% fluorometholone for 6 months. For patients who suffered fungal or acanthamoeba keratitis, administration of the glucocorticoid was terminated 2 weeks after the surgery. Afterwards, immunosuppressants, including cyclosporine A and tacrolimus, were applied topically.

### 2.4. Statistical Analysis

Descriptive statistics were applied, with means and standard deviations (SDs) or numbers and percentages reported where appropriate. Characteristics including age, gender, initial causative organism, initial corneal perforation, initial ocular comorbidities, and initial surgical procedure were compared between patients who received repeat keratoplasty and those who did not, using Fish's exact test (for the abovementioned variables except for age) or *t*-test (for age). Multivariate logistic regression analysis was performed to examine whether the abovementioned characteristics were associated with repeat keratoplasty. Statistical analyses were performed using STATA version 15.0 (Stata Corporation, College Station, TX, USA). A *P* value <0.05 was considered statistically significant.

## 3. Results

A total of 447 patients were identified to receive therapeutic keratoplasty for microbial keratitis. The mean age was 48.7 ± 15.5 years and 290 (64.9%) were male. The most common causative organism of the initial keratitis was fungi (214/447, 47.9%), followed by 160 (35.8%) cases who were cultural-negative (Table 1). The mean diameter of the infectious lesion was 6.6 ± 3.3 mm (data were missing in 122 patients). Data on the depth of the infectious lesion were available in 206 patients. Among them, 29 (14.1%) cases had full-thickness corneal lesion. The number of cases having the lesion in the superficial and deep stroma was 51 (24.8%) and 126 (61.2%), respectively. Corneal perforation was present in 162 (36.2%) patients. Penetrating keratoplasty was performed in about three quarters (337/447) of the cases.

Out of the whole 447 patients, 18 (4.0%) underwent repeat keratoplasty. Their mean age was 45.9 ± 11.3 years and 14 (77.8%) were male. Repeat-to-initial keratoplasty ratio by patient initial causative organism, presence of corneal perforation, and surgical procedure is demonstrated in Table 1. Patients who received repeat keratoplasty were mostly (8/18, 44.4%) cultural-negative for their initial keratitis. Otherwise, fungi (6/18, 33.3%) was the leading causative organism (Table 1).

**Figure 1:**
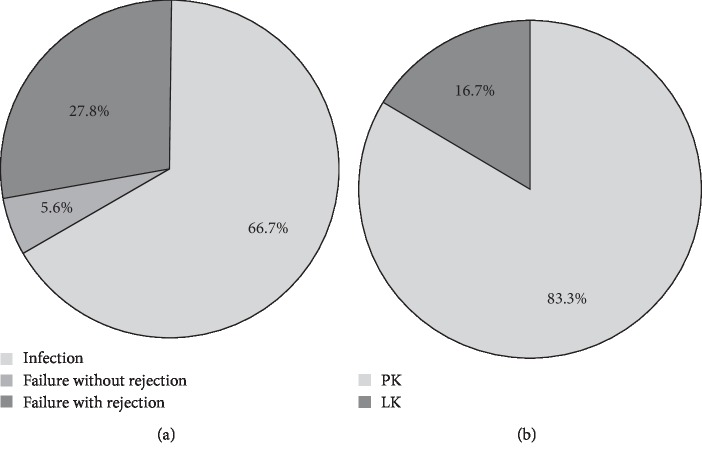
Indications and surgical procedures of repeat keratoplasty for failed therapeutic keratoplasty for microbial keratitis. PK, penetrating keratoplasty; LK, lamellar keratoplasty.

**Figure 2:**
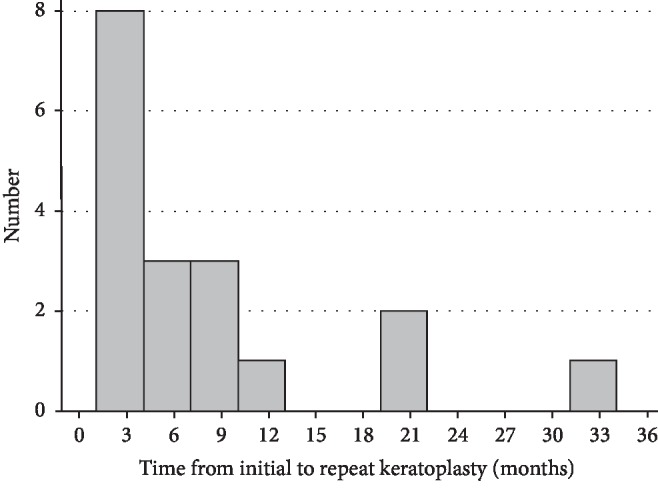
Distribution of the time interval from the initial therapeutic keratoplasty to repeat keratoplasty.

Figure 1 shows the indications and surgical procedures of repeat keratoplasty performed for failed therapeutic keratoplasty. The most common indication was infectious keratitis, accounting for two-thirds (12/18) of the cases. Microbiological tests revealed 3 cases infected by bacteria, 3 cases by fungi, and 1 case by acanthamoeba spp. Another 5 infectious cases were clinically diagnosed but cultural-negative. For the remaining 6 patients who had failed therapeutic keratoplasty and received a second graft, 5 experienced graft rejection and 1 had graft opacity without rejection. Penetrating keratoplasty was performed in 15 (83.3%) patients and lamellar keratoplasty was applied in 3 (16.7%) patients. Distribution of the time interval from initial to repeat keratoplasty is presented in Figure 2. Most of the patients (15/18, 83.3%) received the second keratoplasty within 12 months after the initial therapeutic keratoplasty. Ten (55.6%) patients received repeat keratoplasty within 6 months and seven (38.9%) within 3 months.

Of the 447 cases, postoperative intraocular pressure (data not available in 16 patients) was 7 to 21 mmHg or normal (finger IOP measurement) in 369 (85.6%) patients, > 21 mmHg or elevated (finger IOP measurement) in 38 (8.8%) patients, and <7 mmHg or reduced (finger IOP measurement) in 24 (5.6%) patients. Antiglaucomatous surgeries were performed in 7 patients (one received peripheral iridotomy, two received cyclophotocoagulation, and four received glaucoma valve implantation). Cataract surgery was performed in 37 patients. Among the whole 447 patients, postoperative visual acuity (data not available in 25 patients) was light perception in 76 (18.0%) patients, hand movement in 155 (36.7%) patients, counting fingers in 123 (29.2%) patients, ≥0.02 to < 0.1 in 36 (8.5%) patients, ≤0.1 to < 0.5 in 31 (7.4%) patients, and equal to 0.5 in one (0.2%) patient. Visual acuity after the initial keratoplasty was not statistically significantly different between patients who received repeat keratoplasty and those who did not (*P* = 0.949).

Potential risk factors of repeat keratoplasty for failed therapeutic keratoplasty, including age, gender, initial causative organism, presence of initial corneal perforation, ocular comorbidities, and surgical procedures, were examined. None were found statistically significantly different between patients with or without repeat keratoplasty (Table 2). None of the factors were found associated with repeat keratoplasty in multivariate logistic regression analysis (all *P* > 0.05).

## 4. Discussion

Repeat keratoplasty is the mainstream of therapy for failed therapeutic keratoplasty for microbial keratitis. Only a few previous studies have investigated repeat keratoplasty for failed therapeutic keratoplasty, and the available literature has mainly focused on the anatomical and functional outcomes of the second surgery [12, 13]. Reports concerning ratio of repeat-to-initial keratoplasty and characteristics of the repeat surgery are scarce [14]. This study reported a 4.0% of repeat-to-initial ratio for therapeutic keratoplasty. The major indication of repeat keratoplasty was medically refractory infectious keratitis. Over 80% of the patients received regrafts within 12 months after the initial keratoplasty and penetrating keratoplasty was performed in most of the cases.

With an increasing number of corneal transplantation being performed worldwide, repeat keratoplasty is now becoming one of the leading indications for corneal transplantation [1, 2, 15, 16]. Acceptable graft survival rate and improvement in visual acuity after regrafts were reported among patients who had optically failed therapeutic keratoplasty [12–14]. However, although graft failure rate was reported to be over 50% for therapeutic keratoplasty [12–14], only 4.0% of patients in our study and 3.0% of patients in a previous study [14] received repeat keratoplasty. The ratio of repeat-to-initial keratoplasty is not only a function of the failure rate of initial grafts, but is also influenced by the willingness to repeat the procedure. The low ratio reflected the caution for performing regrafts in patients who had underwent therapeutic keratoplasty. These patients usually had a poorer regraft survival than those with other indications (such as keratoconus and stromal dystrophies) [14], and also a poorer prognosis of the regraft than the initial graft [17]. Moreover, the fact that many patients were on the waiting list for initial keratoplasty might be another reason for the low ratio reported in the current study.

It is well known that patients who have underwent therapeutic keratoplasty possess great preoperative risks for repeat keratoplasty, including peripheral anterior synechiae, posterior synechiae, pupillary membranes, complicated cataract, and a preponderance for glaucoma. These challenges might explain the findings on the indications of repeat keratoplasty in the current study–the majority of the cases who had underwent therapeutic keratoplasty received regrafts for refractory infectious keratitis and only one patient for optically failed graft to improve the vision.

Time interval between the first and the subsequent keratoplasties is another important concern for repeat keratoplasty. Regrafts that are performed too early would increase the risk of graft rejection and failure, while prolonged initial-to-repeat time interval might compromise the clarity of the first graft and influence the type of the second surgical procedure. Since most of the cases received regrafts for refractory infections in the current study, the time intervals between the initial and repeat keratoplasties should be mostly passively determined by the course of the infection and infection control, but not electively for the optical purpose.

We compared the features of patients who received and not received repeat keratoplasty in order to identify possible risk factors of regrafts for therapeutic keratoplasty. Factors including age, gender, initial causative organism, presence of initial corneal perforation, ocular comorbidities, and surgical procedures were examined. None was found of statistically significant impact. Relatively small sample size of patients who underwent repeat keratoplasty may be a reason for the insignificant findings. Moreover, other potentially relevant factors might have been failed to be examined in the current study. It has been reported that the size of the initial therapeutic graft was a significant risk factor that would affect graft survival [18]. Future studies with greater sample size and more detailed data are warranted to reveal significant risk factors for repeat keratoplasty among patients with therapeutic keratoplasty for microbial keratitis.

The current study has all the limitations and potential bias of a retrospective study. However, it is not easy to obtain adequate number of eligible patients using a prospective and population-based design. Data on characteristics of the patients and initial surgery, such as the size of initial therapeutic graft, presence of corneal neovascularization, and presence of peripheral anterior synechiae, were not well documented, degrading the capacity of the current study to identify potential risk factors of repeat keratoplasty for failed therapeutic keratoplasty. It should be noted that a number of different surgeons performed the keratoplasty in the current study. Keratoplasties performed by different surgeons might be argued to have different outcomes. However, the seven surgeons in the present study were all experienced specialists in corneal disorders and well-trained for routine keratoplasty. The probability of influence on the surgical outcomes was minimal.

In conclusion, the ratio of repeat-to-initial keratoplasty among patients who had underwent therapeutic keratoplasty in Southern China was low. The most common indication of repeat keratoplasty was refractory infectious keratitis. Risk factors of regrafts among such patients were not found in the current study. Further investigations with greater sample size and more detailed data on clinical characteristics are needed to reveal potential factors relevant to a secondary keratoplasty after therapeutic keratoplasty for microbial keratitis. Considering the high failure rate of the initial graft of therapeutic keratoplasty, the findings of the current study indicate that repeat keratoplasty for failed therapeutic keratoplasty is cautiously performed in clinical practice. Novel approaches that are potential of increasing the success of the initial and second grafts are warranted.

## Figures and Tables

**Table 1:** Characteristics of initial and repeat keratoplasty.

	Initial keratoplasty		Repeat keratoplasty		Repeat/initial keratoplasty (%)
	Number	Relative contribution (%)	Number	Relative contribution (%)	
Total	447		18		4.0

Initial causative organism					
Bacteria	34	7.6	2	11.1	5.9
Fungi	214	47.9	6	33.3	2.8
Virus	10	2.2	0	0	0
Acanthamoeba	12	2.7	1	5.6	8.3
Mixed	17	3.8	1	5.6	5.9
Cultural-negative	160	35.8	8	44.4	5.0

Initial perforation					
Absent	285	63.8	14	77.8	4.9
Present	162	36.2	4	22.2	2.5

Initial surgical procedure					
PK	337	75.4	12	66.7	3.6
LK	110	24.6	6	33.3	5.5

PK, penetrating keratoplasty; LK, lamellar keratoplasty.

**Table 2:** Demographic and clinical characteristics of patients who underwent therapeutic keratoplasty with and without repeat keratoplasty.

	With repeat keratoplasty (*n* = 18)	Without repeat keratoplasty (*n* = 429)	*P*
Age, years	45.1 ± 11.4	48.8 ± 15.6	0.9937
Gender, *n* of male (%)	14 (77.8)	276 (64.3)	0.317
Initial causative organism, *n* of cases (%)			
Bacteria	2 (11.1)	32 (7.5)	0.459
Fungi	6 (33.3)	208 (48.5)	
Viral	0	10 (2.3)	
Acanthamoeba	1 (5.6)	11 (2.6)	
Mixed	1 (5.6)	16 (3.7)	
Cultural-negative	8 (44.4)	152 (35.4)	

Initial perforation, *n* of cases (%)			
Absent	14 (77.8)	271 (63.2)	0.316
Present	4 (22.2)	158 (36.8)	

Initial ocular comorbidities, *n* of cases (%)			
Absent	16 (88.9)	367 (85.5)	0.577
Chemical burn	1 (5.6)	7 (1.6)	
Ocular trauma	0	20 (4.7)	
Ocular surface disease	1 (5.6)	29 (6.8)	
Glaucoma	0	6 (1.4)	

Initial surgical procedure, *n* of cases (%)			
PK	12 (66.7)	325 (75.8)	0.404
LK	6 (33.3)	104 (24.2)	

PK, penetrating keratoplasty; LK, lamellar keratoplasty.

## Data Availability

The data used to support the findings of this study are available from the corresponding author upon request.
